# Interactions of Multiple Atmospheric Circulation Drive the Drought in Tarim River Basin

**DOI:** 10.1038/srep26470

**Published:** 2016-05-20

**Authors:** Yong-Ping Wu, Guo-Lin Feng, Bai-Lian Li

**Affiliations:** 1 College of Physics Science and Technology, Yangzhou University, Yangzhou, Jiangsu 225002, China; 2 Ecological Complexity and Modeling Laboratory, University of California, Riverside, CA 92521-0124, USA; 3 Zhuhai Joint Innovative Center for Climate-Environment-Ecosystem, Zhuhai Key Laboratory of Dynamics Urban Climate and Ecology, Future Earth Research Institute, Beijing Normal University, Zhuhai 519087, China

## Abstract

Global warming is likely to cause overall drying of land surfaces and aridity increasing leading to expansion of dry climate zones. There is an increased risk of extremely arid environment and large deserts developed progressively in the central Asia. However, the key factors causing the drying in mid-Asia remain inconclusive. Here, we analyzed the relationship among precipitation, water vapor transportation in Tarim River Basin (TRB) and Multiple Atmospheric Circulation (MAC) to explore the mechanism of MAC driving the drying in TRB, through comparing MAC between abundant and scarce precipitation years. We found that Westerly Circulation (WC) and Asian Summer Monsoon (ASM) are likely to promote the precipitation respectively. Whereas, they not only have their own influence but also restrict each other and facilitate the forming of peculiar water vapor transport channel for TRB, which is probably to restrain the precipitation and its distribution pattern and accelerate the drying in this region. Our results enrich the findings on mechanisms of wet places becoming wetter while dry areas getting drier under the global warming.

Decreasing precipitation is inducing the emergence of drought in land surfaces and enlarging dry climate regions as a result of global warming^1^. The middle of Asia is suffering high risk of extreme drought and degenerating into deserts^2^,^3^,^4^,which is leading the deterioration of ecological environment^5^. TRB locates in the center of Asia, which situates in the Northern mid-latitudes area controlled by WC and at the edge of ASM. In addition, TRB is surrounded by a U-shaped topography comprised of Pamirs in the west, Kunlun and Tianshan Mountains in the south and north, and Lop Nur depression in the east. Owing to its unique geographical location and terrain features, the factors of MAC impacting precipitation in this area have become really complex.

Previous studies have shown that there are two major sources of precipitation on land: the water vapor transportation from surrounding areas to inland areas by the movement of the atmosphere, and the local evaporation and water vapor produced by transpiration^6^. The former is related to atmospheric circulation in the horizontal direction, which is more important to the precipitation of inland arid and semi-arid regions such as TRB^7^,^8^. Many researches have analyzed the effects of different scale atmospheric circulation on precipitation^9^. Precipitation over East Africa is associated with a west-east propagating wave of convective activity^10^. Large-scale circulation changes is a direct result of the reduction of precipitation in Northwest Europe; While in southern Europe, its effect is weak when using large-scale circulation to do aggregating simulation about average precipitation^11^. In the central region of Southwest Asia, El Ni
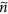
o/Southern Oscillation (ENSO) will also affect local climate, such as in a small area of Northern Pakistan, Afghanistan, Tajikistan, and South of Uzbekistan^12^. The increased precipitation in East China is related to the main features of the atmospheric circulation^13^. It should be noted that some studies pointed out the mid-latitude atmospheric circulation is important because of transporting moisture from the east into the TRB in warm seasons after 1986^14^, but other studies consider water vapor transportation from the west provides the basic source of water vapor for most of the Northwest China^15^. One question that how MAC affects precipitation naturally arises.

When we separated precipitation in TRB to mountains and plains, we found that the amount of and decadal trend in precipitation and the factors over mountains were different from that over plains, the increasing in mountain precipitations were resulted from the forest pump and the accelerating of local water recycling, and the decrease in plain precipitations, the mainly reason of the drying in TRB, is related with MAC.

In this paper, we define MAC as the air movement to and from or around something in a closed/quasi-closed system with different spatial and temporal scales, such as general circulation on planetary scale, *u* wind (zonal wind), Westerly Circulation, Asian Summer Monsoon and so on. And Westerly Circulation (WC) is defined as the prevailing west-to-east winds in westerly belt located in the planetary wind belt between subtropical high pressure zone and subpolar low pressure belt (roughly between latitudes 30~60°N). Since the main factor behind the drying in TRB is the decreasing of plain precipitations^16^, we examined the relationships between any two of MAC, plain precipitations and water vapor flux, analyzed the impact of MAC on them, compared abundant and scare precipitation years, and revealed physical mechanism of MAC driving drought in TRB.

## Results

### Drying and seasonality of precipitation and water vapor flux in TRB

**Figure 1:**
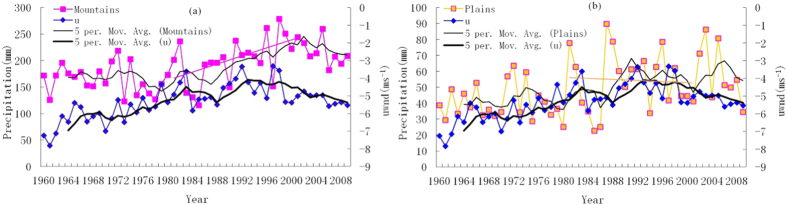
The annual changes and 5-year moving average curve of summer precipitation in mountains (a) and plains (b) of TRB and *u* wind in 600 hPa over Equatorial eastern Pacific. The variations in plain precipitations was fairly consistent with *u* wind in 600 hPa, compared to mountains. Especially, during later 1970s to the beginning of 2000s, precipitation in plains decreased smoothly, although precipitation in mountains increased rapidly. Daily precipitation data of 25 weather stations in TRB of China provided by Cina Meteorological Administration, National Climate Center.

The scarce or zero precipitation is one of the key factors leading to drought. From later 1970s to the beginning of 21st century global warming was significant, although precipitation in mountains increased rapidly owing to the accelerating local water recycling^17^, precipitation in plains showed an smoothly decreasing trend, as well as the trend of the average annual precipitation in total TRB. Besides, comparing Fig. 1a,b, the variations in *u* wind in 600 hPa over Equatorial eastern Pacific was more consistent with precipitation in plains than mountains. It is implied that the decreasing precipitation in plains of TRB is the main factor behind the local drying, the MAC may be the culprit, and water vapor transportation, the joint between precipitation and MAC, should be considered.

**Figure 2:**
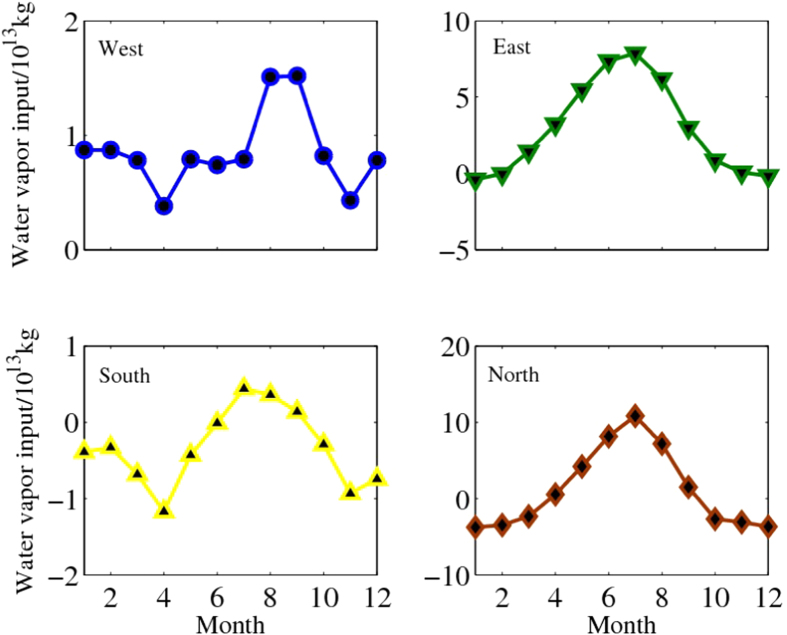
Annual water vapor transportation curve in the four boundary of Tarim River. Seasonal changes in the water vapor transportation from western boundary is much less than the other borders and its amount is obviously weakened during the period of ASM outbreak and development from March to June, but rebound when ASM starts to retreat in August.

Our examinations revealed that the changes of precipitation in TRB was complex and has closed linking with water vapor transportation. Firstly, the spatial distribution of average annual precipitation in TRB presented a pattern of decreasing gradually from northwest to southeast with a seasonality changes, and this was consistent with the direction of the source of water vapor transportation. The seasonality character could be reflected as follows: the most of precipitation occurred in the west-northwest of the TRB in springs, and the water vapor transportation basically came from the west and northwest. In summers, the center of precipitation moved to the north TRB with the entire distribution pattern decreasing from north to south, and the same change took in the water vapor transportation, plenty of water vapor entered into the basin across the Tianshan Mountains from the north and east boundaries. In autumns, the patterns of precipitation and water vapor transportation returned to that in springs; The direction of winter precipitation distribution from strong to weak and direction of water vapor transportation have shifted into west-east. Secondly, the highest precipitation reached 160 mm in Tianshan Mountains in summer, whereas the secondary maxima is only 60 mm in spring. And the water vapor entered into TRB was also mainly from the northern and eastern boundary in summer. Finally, the net input of water vapor from the western boundary is positive throughout the year, while for other boundaries, it is positive in summer and negative in winter. However, the maximum water vapor net input is in summer from east and north. In addition, seasonal changes in the water vapor transportation from western boundary is much less than the other borders. The seasonal characteristics indicated that the channel of TRB water vapor transportation determines the spatial-temporal distribution of precipitation in the region, this implies that both of precipitation and water vapor transportation are closely related to WC and ASM (Fig. 2).

### The coordination of atmospheric circulation in different levels controls precipitation in TRB

**Figure 3:**
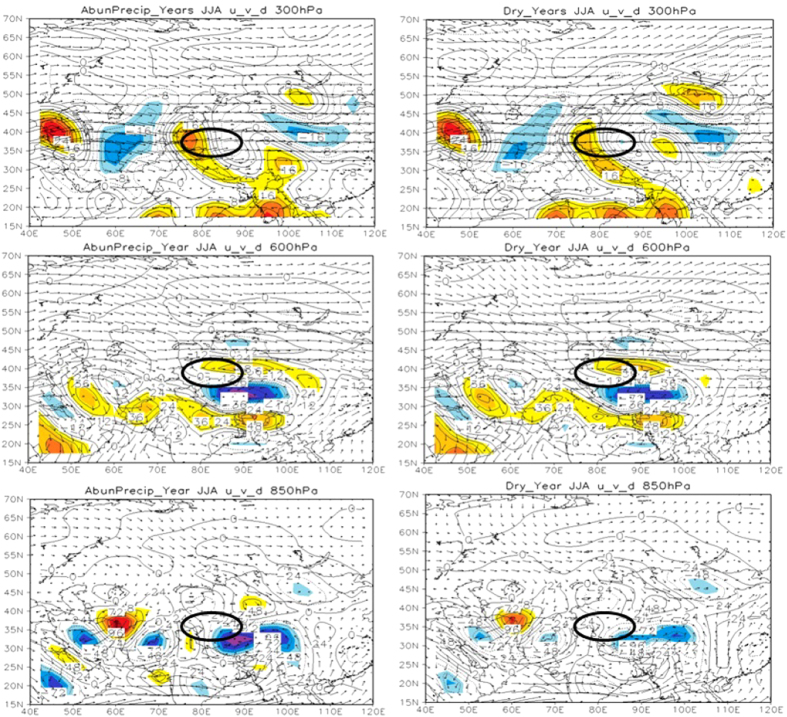
The vector and divergence fields of general circulation in summer in 300 hPa, 600 hPa and 850 hPa in abundant precipitation (dry) years over TRB. NCEP/NCAR monthly mean reanalysis data provided by Earth Research Laboratory (http://www.esrl.noaa.gov/psd/data/gridded/data.ncep.reanalysis.pressure.html). We choose the years with abundant (scarce) precipitation according to the annual precipitation is in the top (bottom) 10%. Years with abundant precipitation are 1974, 1981, 1987, 1996 and 2002, and years with scarce precipitation are 1961, 1970, 1980, 1985 and 1986. We created the vector and divergence fields of general circulation using Grid Analysis and Display System Version 2 (GrADS2.0) and GrADS has been implemented worldwide on a variety of commonly used operating systems and is freely distributed over the Internet (http://badc.nerc.ac.uk/help/software/grads/).

**Figure 4:**
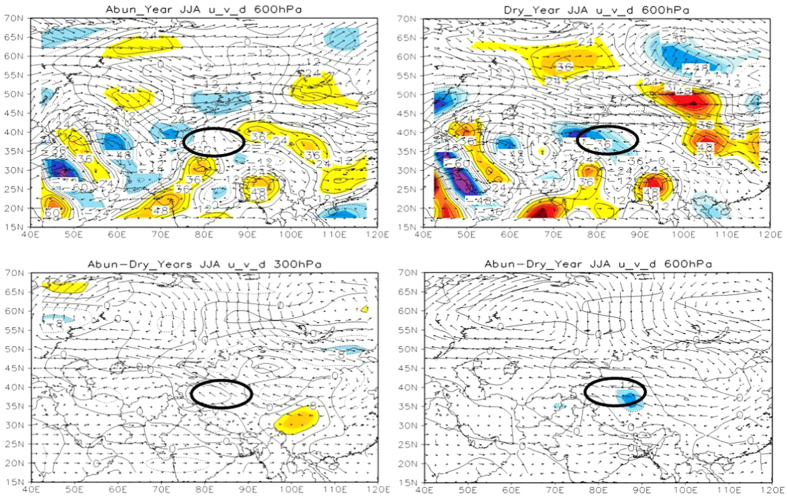
The vector and divergence fields of general circulation of summer in 600 hPa in abundant precipitation year (1996) and dry year (1985) over TRB. The vector and divergence fields of general circulation about the difference between abundant precipitation years and dry years in summer in 300 hPa, 600 hPa over TRB. Monthly mean reanalysis data provided by Earth Research Laboratory (http://www.esrl.noaa.gov/psd/data/gridded/data.ncep.reanalysis.pressure.html). The method for generation of the figure is the same as that in Fig. 3.

To further explore the impact of MAC on TRB precipitation, we characterized the summer atmospheric circulation and its divergence field over TRB in upper (300 hPa for the vector and divergence fields, 200 hPa for *u* wind), middle (600 hPa) and lower (850 hPa) troposphere, and analyzed their correlation with TRB precipitation by comparing the difference between abundant and rare precipitation years. The results showed that TRB was at the water vapor convergence area in 300 hPa and 850 hPa (Fig. 3), in both abundant and rare precipitation years. However, TRB is in front of the westerly trough at 600 hPa in abundant precipitation year, while in a flat westerly circulation in dry year. Compared with the dry years, the water vapor entering into TRB is mainly from the northern and eastern boundary in abundant precipitation years (Fig. 4). It is indicated that the precipitation of TRB is related to the circulation configuration of different levels.

**Figure 5:**
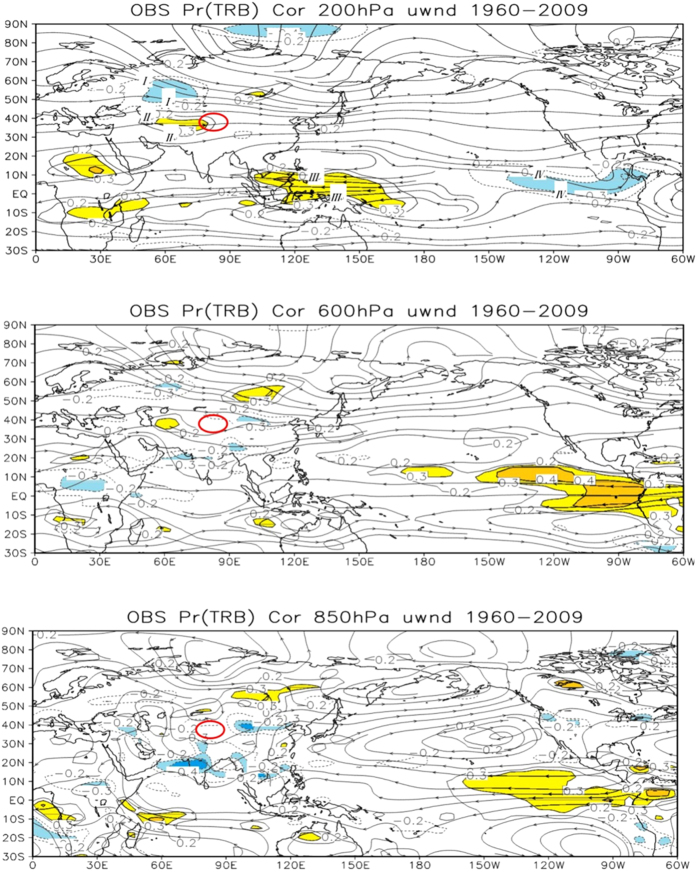
The distribution field of correlation coefficient between precipitation in TRB and uwnd over 200 hPa, 600 hPa and 850 hPa. Shaded area is the significant correlation zone through the 0.05 level Reliability test. There were four significantly high correlation regions: I, II, III and IV. Monthly mean reanalysis data provided by Earth Research Laboratory (http://www.esrl.noaa.gov/psd/data/gridded/data.ncep.reanalysis.pressure.html). The method for generation of the figure is the same as that in Fig. 3.

Correlation analysis between TRB precipitation and zonal wind showed that there were four significantly high correlation regions (*P* > 0.05), which located at I (65°E, 55°N), II (65°E, 45°N), III (135°E, 0°N) and IV region (110°W, 10°N) at 200 hPa (Fig. 5). In area I, which locates at the north to the TRB, there is a significant negative correlation between *u* wind and TRB precipitation. It implies that the weakness of WC there is beneficial to TRB precipitation. Area II is at the entrance of WC to the TRB, and there is a notable positive correlation between *u* wind and TRB precipitation there, which suggests that more water vapor can transport from the Atlantic to the TRB region when the WC is strengthened. Regions III and IV situate respectively at the western equatorial Pacific warm pool and subtropical Eastern Pacific, where El Ni
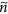
o and the aerial support of Walker Circulation occur. The positive correlation between *u* wind and precipitation in TRB over the western Pacific suggests that the weakening of WC over tropical eastern Pacific Ocean might lead to the reducing of precipitation in TRB. Consequently, it is concluded that the significantly correlated areas over equatorial Pacific Ocean reflects the impact of the ASM and associated sea surface temperature on precipitation of TRB.

Comparative analysis shows that the correlation between *u* wind and TRB precipitation was weaker on 600 hPa and 850 hPa than 300 hPa in I and II zones. While in eastern Mongolia and Qingzang-Tibet Plateau, the correlation is strengthened, with a shift from negative (positive) to positive (negative) in northern (southern) parts. It suggests that the strengthening of upper WC in TRB upstream region or the weakening of WC in middle and lower downstream are benefit to the precipitation of this region. Moreover, in IV region, correlation coefficient is negative in upper troposphere but positive in the middle and lower. It is likely that their different situations in the upper and lower branch of walker circulation cause the opposite correlation with TRB precipitation (Fig. 5).

### The restriction between WC and ASM drives the drying in TRB

The relationship between the situation of precipitation and water vapor flux implies that the decreasing precipitation and drying in TRB is linked to the interaction between WC and ASM. Correlation analysis in Table 1 shows that there is significant positive correlation (*P* > 0.001) between the WC index in middle and whole layer and net input amount of water vapor flowing through west boundary into TRB. While the water vapor net input from east has negative correlation with the lower WC index (*P* > 0.005). In a word, the correlation of WC index with zonal water vapor net input is closer than that with meridional water vapor net input (Table 1). We can conclude that strengthened WC will enrich the water vapor flowing into TRB across west boundary, but weaken that from east boundary.

**Figure 6:**
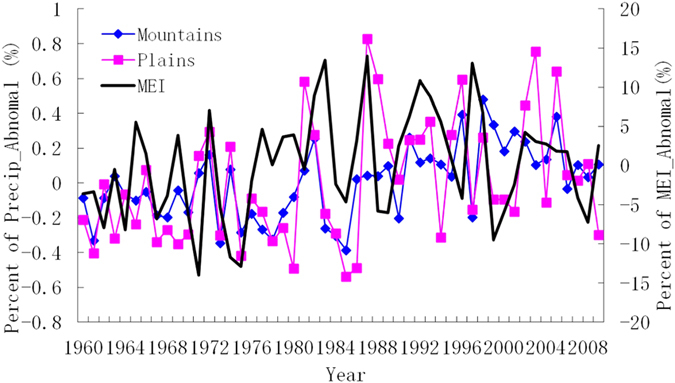
Inter-annual variation in anomaly percentage of Multivariate ENSO Index (MEI) and the precipitation in mountains and plains over TRB. Both interdecadal trends and interannual shock in MEI were more consistent with that in plains than mountains.

Due to convective activity, the link between ASM and ENSO is close^18^. ENSO event has a significant impact on China precipitation and associated atmospheric circulation^19^,^20^. Syed *et al.* pointed out that ENSO also affect local climate in the central region of Southwest Asia and found positive precipitation anomalies accompanying with warm ENSO^21^. Plenty of studies insist ENSO takes some impact on China and regional precipitation. In this paper, the relationship between ENSO and the precipitation in TRB was comparatively analyzed, using Multivariate ENSO Index (MEI) established by Wolter and Timlin^22^. Statistics analysis showed that MEI has a positive significant correlation with the water vapor net input cross western (W_WVNI), but negative notable correlations with that through eastern (E_WVNI), northern (N_WVNI) boundaries of the TRB, and the meridional water vapor net input (M_WVNI) (Fig. 6 and Table 2). Since there was a significant negative correlation between ASM with the same period ENSO after 1980^23^, ASM is not conducive to water vapor entering the TRB from western border but eastern and northern borders.

In summary, strengthened WC is likely to increase water vapor net input to TRB from west boundary, and the reinforced ASM is benefit to moisture flowing into TRB cross east boundary. However, their redoubling simultaneously will restrict each other. In fact, western water vapor net input is obviously weakened during the period of ASM outbreak and development from March to June, but rebound when ASM starts to retreat in August (Fig. 2). Therefore, the competition between WC and ASM will reduce the precipitation and induce drying in the area.

## Discussion

Empirical results revealed that the central Asia is undergoing an increased risk of extremely arid environment. However, the underlying causes of this phenomenon are far from being well understood. To reveal a mechanism of it, we take TRB as a typical example to analyze how precipitation, water vapor transportation and MAC have influence on drying. It is found that interactions of WC and ASM decrease the amount of precipitation and thus promote the emergence the drying. Our findings well enrich the mechanisms of wet places becoming wetter while dry areas getting drier under the environment of global warming^24^,^25^,^26^.

It should be noted that our results are obtained based on data analysis. However, for a long time prediction on climate change, data analysis is not very effective due to requiring large amounts of data. In this case, it may provide useful information by constructing mathematical models to explain the phenomenon observation or make the forecast of climate change. As a result, we need to investigate climate change based on both mathematical analysis and data processing in the future work.

In this paper, we just focus our attention on the region of TRB. However, it is believed that the findings may be also widely applicable in other regions. Two main reasons may support our conclusion. Firstly, from a geographical point of view, some regions have similar geographical environments with TRB, such as Sahara Desert^27^. Secondly, from the data point of view, the data in some areas exhibit same features with TRB^28^,^29^. We hope that our efforts will provide a new starting point for the study of drying causes in modeling^30^,^31^.

## Method

### The computation of water vapor transportation and water vapor net input (WVNI)

A rectangular box was chosen to investigate the budget for the water vapor flux. The vertically integrated water vapor flux *Q* can be expressed as^17^.


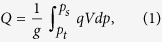


where *g* is the gravitational acceleration, *P*_*s*_ is the surface pressure, *P*_*t*_ is pressure at the top of the air column taken as 300 hPa, *q* is the specific humidity, *V* is the wind velocity, and *p* is the atmospheric pressure. The zonal and meridional components of *Q* denoted by *Q*_*x*_ and *Q*_*y*_, are expressed respectively as:


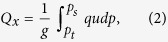



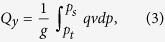


where *u* and *v* are zonal and meridional wind respectively. When *Q*_*x*_ of western boundary or *Q*_*y*_ of southern boundary is positive, or *Q*_*x*_ of eastern boundary or *Q*_*y*_ of northern boundary is negative, it will be indicated that there is water vapor transported into TRB. Otherwise, there is water vapor transported out of TRB. The zonal component of water vapor net input (WVNI) is equal to the results of water vapor flux of western boundary subtracted by that of eastern boundary. And the meridional component is equal to the southern boundary minus the northern boundary. NCEP/NCAR monthly mean reanalysis data (2.5° × 2.5°) provided by Earth Research Laboratory (http://www.esrl.noaa.gov/psd/data/gridded/data.ncep.reanalysis.pressure.html). Su *et al.*^32^ comprehensively tested the NCAR/NCEP reanalysis data set in China, especially the Tibetan Plateau and adjacent areas, and pointed out that the patterns of meteorological elements (including temperature, pressure, *u*/*v* wind, humidity and precipitation) of reanalysis data are similar as those of climate analysis.

### Estimate of the air divergence

The physical meaning of air divergence is the air quality which converges in or diverges out of per unit volume during per unit time. The horizontal air divergence *A* is:


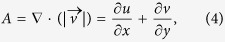


where, *u* and *v* are zonal and meridional wind velocity respectively. When *A* > 0, it is divergence; when *A* < 0, it is convergence. The unit is 1/s (inverse seconds).

### Calculations of MEI for ENSO

The ENSO is one of the Earth’s strongest climate vibration on inter-annual time scales and has global influences. The Multivariate ENSO Index (MEI) established by Wolter and Timlin is considered as the most representative since it links six different meteorological parameters measured over the tropical Pacific^33^,^34^.

### Calculations of WCI

WCI is calculated according to 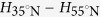
35, where *H* is the average height in 500 hPa from 40°E to 100°E.

Besides, the R/S^36^, Mann Kendall (MK)^37^ mutation analysis, and linear trend analysis are used to analyze the mutation time and changing trends of net input of water vapor, precipitation and other relevant factors.

## Additional Information

**How to cite this article**: Wu, Y.-P. *et al.* Interactions of Multiple Atmospheric Circulation Drive the Drought in Tarim River Basin. *Sci. Rep.* **6**, 26470; doi: 10.1038/srep26470 (2016).

## Figures and Tables

**Table 1:** Correlation coefficient between water vapor net input (WVNI) and WC index, where H is the average height in 500 hPa from 40E to 100E.) in upper, middle, lower and the whole troposphere for 1960–2009 over TRB.

	Upper	Middle	Lower	Whole layer
*WCI* − *Z*	0.166	0.069	0.029	0.111
*WCI* − *E*	−0.037	−0.109	−0.211	−0.138
*WCI* − *Z*	0.157	−0.137	−0.337^a^	−0.231
*WCI* − *W*	0.078	0.489^b^	/	0.402^a^

Z and M are WVNI in Zonal and Meridian, E and W represent WVNI from east and west boundaries respectively.

^a^*P* > 0.02.

^b^*P* > 0.001.

**Table 2:** Correlation coefficient between Multivariate ENSO Index and water vapor net input through west (W_WVNI), east (W_WNI) and north (N_WVNI) boundaries and the meridional water vapor net input (M_WVNI) for 1970–2009 over TRB.

	W_WVNI	E_WVNI	N_WVNI	M_WVNI
MEI	0.380^a^	−0.268^b^	−0.288^b^	−0.262^a^

^a^*P* > 0.01.

^b^*P* > 0.1.
